# Causal Effects of Circulating Lipid Traits on Epithelial Ovarian Cancer: A Two-Sample Mendelian Randomization Study

**DOI:** 10.3390/metabo12121175

**Published:** 2022-11-25

**Authors:** Hongen Meng, Rong Wang, Zijun Song, Fudi Wang

**Affiliations:** 1 The Fourth Affiliated Hospital, The First Affiliated Hospital, Institute of Translational Medicine, School of Public Health, Cancer Center, State Key Laboratory of Experimental Hematology, Zhejiang University School of Medicine, Hangzhou 310030, China; 2 Liangzhu Laboratory, Zhejiang University Medical Center, 1369 West Wenyi Road, Hangzhou 311121, China; 3 The First Affiliated Hospital, Basic Medical Sciences, School of Public Health, Hengyang Medical School, University of South China, Hengyang 421001, China

**Keywords:** circulating lipid, apolipoprotein, triglyceride, Mendelian randomization, epithelial ovarian cancer

## Abstract

Ovarian cancer (OC), and particularly epithelial OC (EOC), is an increasing challenge for women. Circulating lipids play different roles in the occurrence and development of OC, but no causal relationship has been confirmed. We used two-sample Mendelian randomization (MR) to evaluate the genetic effects of circulating Apolipoprotein A1 (APOA1), Apolipoprotein B (APOB), high-density lipoprotein (HDL) cholesterol, low-density lipoprotein (LDL) cholesterol, and triglyc-erides (TG) on EOC risks based on summary data obtained from the UK Biobank and the Ovarian Cancer Association Consortium. We used the inverse-variance weight as the main statistical method and the MR-Egger, weighted median, and MR-PRESSO for sensitivity analysis. A 1-SD increment in HDL gave odds ratios (OR) and 95% confidence intervals (CI) of OR = 0.80 (95% CI: 0.69–0.93), OR = 0.77 (95% CI: 0.66–0.90), and OR = 0.76 (95% CI: 0.63–0.90) for low malignant potential OC (LMPOC), low-grade low malignant OC (LGLMSOC), and low malignant serous OC (LMSOC), respectively. Genetic liability due to TG was associated with an increased risk of LGLMSOC and LGSOC and a suggestive association with an increased risk of LMSOC (*p* = 0.001, *p* = 0.007, and *p* = 0.027, respectively). Circulating HDL was negatively associated with the risk of LMPOC, LGLMSOC, and LMSOC, while elevated circulating TG levels genetically predicted an increased risk of LGLMSOC and LGSOC. Further research is needed to investigate the causal effects of lipids on EOC and potential intervention and therapeutic targets.

## 1. Introduction

Ovarian cancer (OC) is a highly heterogeneous gynecological malignancy that accounted for approximately 185,000 deaths and 295,400 diagnoses in women in 2018 [1]. The Global Cancer Observatory predicts 434,184 cases of OC globally in 2040, an increase of approximately 50% [2]. The most ubiquitous type of OC is epithelial ovarian cancer (EOC) (over 95% of all OC). According to the natural factors of pathogenesis, gene expression, prognosis, and other risk factors, EOC is further divided into five histologic subtypes: the most common histologic subtype high-grade serous (HGSOC) (70%), followed by clear cell (10%), endometrioid (10%), low-grade serous (LGSOC) (<5%), and mucinous (3%) [3]. Most newly identified instances of OC are already in an advanced state due to a lack of early identifiable clinical symptoms, precise laboratory markers, and efficient screening methods [4]. OC is a leading cause of death in women (47% 5-year survival) [5]; therefore, the early identification, intervention, and management of ovarian malignancies remain a global challenge.

A poor understanding of the etiology and risk factors for the initiation and progression of OC has hampered its intervention and effective therapy. Known risk factors include menarche age, natural menopause age, and endometriosis age [6]; moreover, modifiable risk factors for OC include cigarette consumption, hormonal substitution treatment, and dietary variables [6,7]. Increased dietary intakes of fiber [8] and soy [3] have shown positive preventive effects against OC. A risk of OC was linked to low levels of vitamin D [9].

In recent years, an association has been documented between OC and circulating lipids in several epidemiological observational studies. One observational cohort study showed that elevated levels of triglyceride (TG) and low high-density lipoproteins (HDL) were significantly associated with a high severity of EOC [10]. Similarly, a meta-analysis study found a link between decreased HDL profiles and OC manifestations and growth [11]. Zhang et al. [12] also showed an association between high HDL levels and a lower ovarian cancer risk, but they found no significant associations between TG and OC. By contrast, Delimaris et al. [13] and Melvin et al. [14] found no association between HDL and OC risk. These conflicting results indicate that circulating lipids might be closely related to OC; however, observational studies are susceptible to potential confounding factors, including small sample sizes, short follow-up durations, and inaccurate classifications of OC.

Given these limitations of observational studies and the growing numbers of datasets of summary statistics from genome-wide association studies (GWAS), we recognized that Mendelian randomization (MR) could be used to investigate the potential causal association between circulating lipids and EOC. Using genetic predisposition as an instrumental variable for exposure which diminishes confounding as genetic variants independent of self-selected lifestyle factors and behaviors, MR was subjected to several sensitivity analyses for the efficient and reliable generation of results based on Mendel’s laws of inheritance. Since genetic variants (alleles) are randomly assorted at meiosis which precedes the onset of disease, this process could uncover the reverse causality biases prevalent in observational studies. In this study, we conducted a two-sample MR study to investigate the association between circulating lipids and EOC based on two recently released large enough and abundant GWAS datasets.

## 2. Experimental Design

### 2.1. Assumptions of MR Study and Study Design Overview

**Figure 1:**
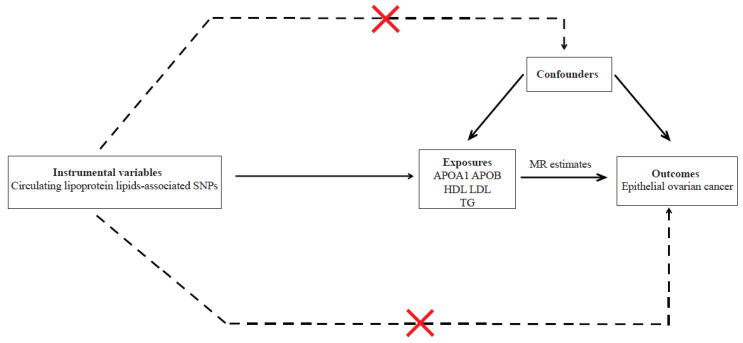
Schematic representation of MR analyses. APOA1, APOB, HDL, LDL, and TG SNPs were used as instrumental variables to investigate the causal effect of lipids on EOC. The arrows indicate the MR assumptions such that the instrumental variable is associated with the exposure—not associated with confounders—and affects the outcome only via the exposure. APOA1, apolipoprotein A1; APOB, apolipoprotein B; HDL, high-density lipoprotein cholesterol; LDL, low-density lipoprotein cholesterol; TG, triglycerides; MR, Mendelian randomization; SNP, single-nucleotide polymorphism.

When performing the MR analysis, three assumptions were observed: (i) relevance assumption, (ii) independent assumption, and (iii) exclusion restriction assumption. The overall study design is illustrated in Figure 1. Summary-level data from the UK Biobank (UKBB) [15] and the Ovarian Cancer Association Consortium (OCAC) [16] was used in this present two-sample MR study. Appropriate patient consent and ethics approval were obtained in the original studies.

### 2.2. Instrumental Variables

Single-nucleotide polymorphisms (SNPs) associated with circulating apolipoprotein A1 (APOA1) (299), apolipoprotein B (APOB) (198), HDL (362), LDL (177), and TG (313) at the genome-wide significance level (*p* < 5 × 10^−8^, linkage disequilibrium (LD) threshold of r^2^ < 0.001 and located 1 Mb apart from each other) were identified from a multivariable MR analysis of GWAS with up to 393,193, 439,214, 403,943, 440,546, and 441,016) separate individuals of European ancestry in the UKBB (Table 1) [15] using R. The mean age of the members was 56.9 y (extend 39–73 y) and 54.2% were women. Detailed information about the GWASs utilized is displayed in Table 1.

### 2.3. Outcome Data Sources

For our outcome data, we used the OCAC dataset, which is a case–control study of EOC that included 25,509 population-based EOC cases and 40,941 controls [16]. In the OCAC study, 12 histotypes were investigated (all EOC, clear cell OC, endometrioid OC, low malignant potential OC (LMPOC), HGSOC, LGSOC, high-grade and low-grade serous OC (HGLGSOC), serous OC: low-grade and low malignant potential (LGLMSOC), serous ovarian cancer: low malignant potential (LMSOC), mucinous ovarian cancer (MOC): invasive and low malignant potential, invasive mucinous ovarian cancer, and low malignant potential mucinous ovarian cancer (LMMOC)). The GWAS was based on the OCAC use of a 1000 Genomes Project reference panel to impute genotypes for 11,403,952 common variants. It evaluated the associations of these SNPs with EOC risks adjusted for study and population substructure by including the eigenvectors of project-specific principal components as covariates in the model. The outcome data were retrieved based on a previously described method [17].

All considerations included within the GWASs had been affirmed by relevant ethical review committees, and all members had provided written informed consent. The current study utilized summary-level information that was freely accessible; in this way, no additional ethical review was required for this research.

### 2.4. Statistical Analysis

The multiplicative random effects inverse-variance weighted (IVW) model was utilized as the main statistical method, and the weighted median [18], MR-Egger [19], and MR-PRESSO [20] were chosen as sensitivity analyses. As 5 exposures were conducted, the adjusted threshold value was *p* < 0.01 (0.05/5). All the MR tests and sensitivity analyses were based on the R packages (two-sample MR [17], MR-PRESSO [20], and Mendelian randomization [21]) and a GWAS summary data library developed as a platform [17,22] using R (version 4.1.1, the R Core team, Boston, MA, USA). All instrument SNPs and related information used in the study are in Supplementary Dataset S1.

## 3. Results

The genetic predisposition to higher HDL was associated with a decreased risk of LMPOC, LGLMSOC, and LMSOC. For an increase in HDL of 1-SD, the odds ratios (OR) and 95% confidence intervals (CI) were OR = 0.80 (95% CI: 0.69–0.93) for low malignant potential OC (LMPOC), OR = 0.77 (95% CI: 0.66–0.90) for low-grade low malignant OC (LGLMSOC), and OR = 0.76 (95% CI: 0.63–0.90) for low malignant serous OC (LMSOC), respectively. These associations remained significant within the sensitivity analysis utilizing the MR-PRESSO strategy after the expulsion of one exception (Table 2), but they did not persist as noteworthy within the weighted median and MR-Egger analyses.

Hereditary risk due to TG appeared as an association with an increased chance of LGLMSOC and LGSOC (*p* = 0.001, *p* = 0.007, separately) (Table 3). The associations remained reliable within the sensitivity analysis utilizing the MR-PRESSO strategy but not the weighted median and MR-Egger strategies.

No significant associations were recognized for APOA1, APOB, and LDL with EOC within the primary examination or within the affectability investigations of each data source (Supplementary Tables S1–S3). No pleiotropy was detected in APOA1 and LDL analysis (all the MR-Egger regression *p* values > 0.05) (Supplementary Tables S1–S3).

## 4. Discussion

The multiple MR sensitivity analyses arrived at a conclusion that serum HDL was negatively associated with the risk of LMPOC, LGLMSOC, and LMSOC, and that TG was positively associated with the risk of LGLMSOC and LGSOC.

The results of epidemiological studies showing an association between HDL and EOC are consistent with our MR analysis. The mixed study conducted by Zhang et al. [12] found an association between high HDL and lower OC risk, in agreement with the finding of an association between HDL and malignant OC by Onwuka JU [11]. Low HDL levels were also shown to correlate with the high severity of EOC [10], while high HDL levels showed a significant association with better progression-free survival (PFS) of EOC patients [23]. The HDL-associated serum paraoxonase activity and arylesterase activity were also significantly lower in patients with EOC than in controls [24]. HDL-mediated lipid transport pathways were associated with PFS and the overall survival of EOC patients in a GWAS study [25]. Nevertheless, no relationship was established between these factors in other studies [13,14]. The reverse causality results from these cohort and meta-analysis studies demonstrate the limitations of small sample sizes and the vague types or classifications due to the heterogeneity of EOC. However, the present analysis, using precisely divided subtypes of EOC, documented a causal association between HDL and EOC.

As with HDL, TG has also been associated with OC. For example, Zhang et al. [10] found a significant association between elevated TG levels and high severity of EOC. Another study found that blood TG levels at clinically relevant cut-off points (>200 vs. ≤200 mg/dL) for cases diagnosed for more than 2 years showed a positive relationship with EOC risk [26]. An increased concentration of TG was also observed in a Japanese EOC study [27]. A prospective analysis found that circulating TG was a risk biomarker for OC, particularly for rapidly fatal tumors [28]. Interestingly, during the generation of highly aggressive EOC cell lines, TG levels were dramatically increased [29]. Some previous articles claimed that TG had no causal effect on EOC; however, the present analysis indicated a clear correlation between elevated TG and an increased risk of LGLMSOC and LGSOC.

A few studies have linked APOA1, APOB, and LDL with EOC. For example, in 2010, Li et al. [30] showed that LDL was an independent predictor of OC survival, which was significantly shorter in patients with elevated LDL among 132 stage IIIC/IV patients. Consistent with that finding, Lin et al. [23] reported a significant association between high LDL levels and worse overall survival in 156 patients with EOC who underwent surgical resection. However, a retrospective study that included 267 cases showed an independent association between increased preoperative LDL levels and improved 5-year recurrence-free survival [31]. Several studies have demonstrated that LDL, APOA1, and APOB showed no significant association with OC [10,11,12,14,23,32]. In the present study, no causal role was found for LDL, APOA1, or APOB regarding the subtypes of EOC.

The underlying mechanisms by which HDL and TG affect the EOC risk remain to be established. HDL promotes inflammation, apoptosis, angiogenesis, immunomodulatory activities, and oxidation to exacerbate cancer development. HDL activates APOA1, LCAT, and others to protect LDL from oxidative modification, thereby confirming its antioxidative properties [33,34]. In the case of EOC, in a mouse model of ovarian epithelial papillary serous adenocarcinoma, the overexpression of human APOA1 not only elevated HDL levels but also hindered tumor development and improved survival rate [35]. Bovine HDL inhibited ovarian tumor development by reducing the accumulation and/or synthesis of pro-inflammatory lipids through a reduction in plasma levels of lysophosphatidic acid [36].

The results of the present study are also supported by experimental data showing a pivotal role of TG in EOC tumorigenesis. TG is the primary fat stored in adipose tissue, but it also causes increases in adipocyte size and number due to fat accumulation when it is present in excess [37]. The omentum, an adipocyte-rich tissue, is the main intraperitoneal site of OC metastasis [38]. The adipocyte-rich microenvironment favors OC metastasis through fatty acid oxidation [39] and salt-inducible kinase 2 (SIK2)-mediated PI3K-AKT cancer cell proliferation/survival [40]. TG metabolism can also participate in ovarian carcinogenesis by providing essential fatty acids [39] or insulin-mediated inflammation by cyclooxygenase-2 (COX-2) [10].

As far as we know, this study is the first to examine the genetic relationships among APOA1, APOB, HDL, LDL, and TG on EOC using the MR analysis method and a two-sample MR approach. MR analyses can minimize potential confounding and reverse causality due to the random allocation of genotypes. In this study, we also employed the most recent and largest datasets from the UKBB and retained only European descent participants to avoid population stratification. The inclusion of these larger datasets was coupled with a rigorous approach (LD < 0.001) for SNP selection. The Bonferroni test (adjusted *p* for association < 0.01) was conducted to increase the precision and the statistical power as much as possible. We also applied multiple methods in MR sensitivity analysis, including MR-Egger, weighted median, and MR-PRESSO, to minimize bias and provide strong causal results. Some epidemiological studies have provided evidence that links the concentration of APOA1, APOB, HDL, LDL, and TG to the risk of OC, but we further analyzed the effect on the subtypes of ovarian cancer to rule out false-positive results.

Our study had several limitations. One is the limited exposures included only APOA1, APOB, HDL, LDL, and TG. More hereditary instrumental variables associated with total cholesterol, free cholesterol, and other lipids should be evaluated. Another limitation is that this study only took European ancestry into account, and this could place restrictions on the inference of findings to other populations. A third limitation is that heterogeneity was not fully avoided, even though most of the results based on IVW were consistent with the results based on MR-PRESSO. A further limitation arose because, although the OCAC dataset was large, the separate subgroup sample size of OC was not sufficiently large. More studies and cases should be included. In any case, our study offers unused pieces of knowledge for the connections between lipids and the hazard of OC, subsequently giving an improved understanding of its etiology.

In conclusion, through multiple analyses based on MR, we found distinct genetic influence patterns for APOA1, APOB, HDL, LDL, and TG on different subtypes of OC. In particular, circulating HDL was negatively associated with the risk of LMPOC, LGLMSOC, and LMSOC, whereas elevated levels of serum TG levels genetically predicted an increased risk of LGLMSOC and LGSOC. Further research is needed to investigate the causes and underlying mechanisms of lipid effects on EOC and to establish potential interventions and therapeutic targets.

## Figures and Tables

**Table 1:** Characteristics of UK Biobank datasets and OCAC.

Exposures	Consortium	No. SNPs	Sample Size	Adjustments	Population
APOA1	UK Biobank	299	393,193	Age, sex, and genotyping chip	European
APOB	UK Biobank	198	439,214		
HDL	UK Biobank	362	403,943		
LDL	UK Biobank	158	440,546		
TG	UK Biobank	313	441,016		
**Main outcomes**	**Dataset**	**No. cases**	**Control**	**Total**	**Population**
All SOC	OCAC	25,509	40,941	66450	European
Clear cell OC	OCAC	1366	40,941		
Endometrioid OC	OCAC	2810	40,941		
LMPOC	OCAC	3103	40,941		
HGLGSOC	OCAC	14,049	40,941		
HGSOC	OCAC	13,037	40,941		
LGSOC	OCAC	1012	40,941		
LGLMSOC	OCAC	2966	40,941		
LMSOC	OCAC	1954	40,941		
Invasive and low malignant potential MOC	OCAC	2566	40,941		
Invasive MOC	OCAC	1417	40,941		
LMMOC	OCAC	1149	40,941		

Abbreviations: APOA1, apolipoprotein A1; APOB, apolipoprotein B; HDL, high-density lipoprotein cholesterol; LDL, low-density lipoprotein cholesterol; TG, triglycerides; SNP, single-nucleotide polymorphism; OC, ovarian cancer; SOC, serous ovarian cancer; MOC, mucinous ovarian cancer; LMPOC, low malignant potential ovarian cancer; HGLGSOC, high-grade and low-grade serous ovarian cancer; HGSOC, high-grade serous ovarian cancer; LGSOC, low-grade serous ovarian cancer; LGLMSOC, serous ovarian cancer: low-grade and low malignant potential; LMSOC, serous ovarian cancer: low malignant potential; LMMOC, low malignant potential mucinous ovarian cancer.

**Table 2:** Two-sample Mendelian randomization estimations showing the effect of HDL on EOC.

Main Outcome	Method	No. of SNPs	OR (95% CI)	*p* for Association	*p* for Heterogeneity Test	*p* for MR-Egger Intercept	*p* for MR-PRESSO Global Test
All EOC	IVW	322	1.02 (0.94–1.10)	0.697	<1 × 10^−3^	0.218	
	MR Egger	322	1.08 (0.95–1.21)	0.235	<1 × 10^−3^		
	Weighted median	322	1.05 (0.94–1.17)	0.376			
	MR-PRESSO (outlier corrected, 2 outliers)	320	1.01 (1.01–1.02)	0.719			<1 × 10^−4^
Clear cell OC	IVW	322	1.20 (0.98–1.46)	0.084	0.093	0.655	
	MR Egger	322	0.96 (0.83–1.55)	0.435	0.088		
	Weighted median	322	1.11 (0.76–1.62)	0.589			
	MR-PRESSO (raw, 0 outliers)	322	1.20 (1.18–1.21)	0.085			0.090
Endometrioid OC	IVW	322	0.98 (0.85–1.14)	0.798	0.041	0.469	
	MR Egger	322	1.05 (0.83–1.31)	0.701	0.040		
	Weighted median	322	1.24 (0.97–1.59)	0.082			
	MR-PRESSO (raw, 0 outliers)	322	0.98 (0.97–0.99)	0.798			0.037
LMPOC	IVW	322	0.80 (0.69–0.93)	0.004	<1 × 10^−3^	0.155	
	MR Egger	322	0.91 (0.72–1.15)	0.439	<1 × 10^−3^		
	Weighted median	322	0.79 (0.63–0.99)	0.039			
	MR-PRESSO (outlier corrected, 1 outlier)	321	0.81 (0.81–0.82)	0.005			<1 × 10^−3^
HGLGSOC	IVW	322	1.00 (0.91–1.10)	0.930	<1 × 10^−3^	0.174	
	MR Egger	322	1.08 (0.94–1.25)	0.276	<1 × 10^−3^		
	Weighted median	322	1.05 (0.93–1.20)	0.429			
	MR-PRESSO (outlier corrected, 3 outliers)	319	1.01 (1.00–1.01)	0.882			<1 × 10^−4^
HGSOC	IVW	322	1.02 (0.92–1.12)	0.738	<1 × 10^−3^	0.224	
	MR Egger	322	1.09 (0.94–1.27)	0.254	<1 × 10^−3^		
	Weighted median	322	1.09 (0.95–1.25)	0.232			
	MR-PRESSO (outlier corrected, 2 outliers)	320	1.01 (1.01–1.02)	0.782			<1 × 10^−4^
LGSOC	IVW	322	0.80 (0.63–1.01)	0.064	0.283		
	MR Egger	322	0.94 (0.66–1.36)	0.756	0.288	0.245	
	Weighted median	322	0.86 (0.58–1.27)	0.440			
	MR-PRESSO (raw, 0 outliers)	322	0.80 (0.79–0.81)	0.065			0.280
LGLMSOC	IVW	322	0.77 (0.66–0.90)	0.001	0.001	0.228	
	MR Egger	322	0.86 (0.68–1.09)	0.221	0.001		
	Weighted median	322	0.84 (0.66–1.07)	0.158			
	MR-PRESSO (outlier corrected, 1 outlier)	321	0.78 (0.78–0.79)	0.001			0.001
LMSOC	IVW	322	0.76 (0.63–0.90)	0.002	0.024	0.358	
	MR Egger	322	0.83 (0.63–1.10)	0.197	0.023		
	Weighted median	322	0.81 (0.62–1.08)	0.152			
	MR-PRESSO (outlier corrected, 1 outlier)	321	0.77 (0.76–0.78)	0.002			0.023
MOC: invasive and low malignant potential	IVW	322	0.98 (0.84–1.15)	0.821	0.023	0.015	
	MR Egger	322	1.23 (0.97–1.55)	0.088	0.037		
	Weighted median	322	1.07 (0.82–1.40)	0.609			
	MR-PRESSO (raw, 0 outliers)	322	0.98 (0.97–0.99)	0.821			0.024
Invasive MOC	IVW	322	1.08 (0.88–1.32)	0.456	0.075	0.029	
	MR Egger	322	1.40 (1.03–0.09)	0.032	0.100		
	Weighted median	322	1.18 (0.83–1.68)	0.361			
	MR-PRESSO (raw, 0 outliers)	322	1.08 (1.07–1.09)	0.457			0.075
LMMOC	IVW	322	0.86 (0.68–1.10)	0.228	0.001	0.194	
	MR Egger	322	1.04 (0.72–1.50)	0.841	0.001		
	Weighted median	322	0.86 (0.59–1.26)	0.446			
	MR-PRESSO (raw, 0 outliers)	322	0.86 (0.85–0.88)	0.229			0.001

Abbreviations: HDL, high-density lipoprotein cholesterol; MR, Mendelian randomization; IVW, inverse-variance weighted; OR, odds ratio; CI, confidence interval; SNP, single-nucleotide polymorphism; OC, ovarian cancer; EOC, epithelial ovarian cancer; SOC, serous ovarian cancer; MOC, mucinous ovarian cancer; LMPOC, low malignant potential ovarian cancer; HGLGSOC, high-grade and low-grade serous ovarian cancer; HGSOC, high-grade serous ovarian cancer; LGSOC, low-grade serous ovarian cancer; LGLMSOC, serous ovarian cancer: low-grade and low malignant potential; LMSOC, serous ovarian cancer: low malignant potential; LMMOC, low malignant potential mucinous ovarian cancer.

**Table 3:** Two-sample Mendelian randomization estimations showing the effect of TG on EOC.

**Main Outcomes**	**Method**	**No. of SNPs**	**OR (95% CI)**	***p*** **for Association**	***p*** **for** **Heterogeneity Test**	***p*** **for MR-Egger** **Intercept**	***p*** **for MR-PRESSO Global Test**
All EOC	IVW	280	1.05 (0.97–1.13)	0.204	<1 × 10^−3^	0.092	
	MR Egger	280	0.98 (0.87–1.09)	0.674	0.001		
	Weighted median	280	0.97 (0.87–1.09)	0.631			
	MR-PRESSO (raw, 0 outliers)	280	1.05 (1.05–1.05)	0.205			<1 × 10^−3^
Clear cell OC	IVW	280	0.88 (0.72–1.08)	0.222	0.272	0.500	
	MR Egger	280	0.81 (0.60–1.11)	0.190	0.265		
	Weighted median	280	0.81 (0.56–1.15)	0.237			
	MR-PRESSO (raw, 0 outliers)	280	0.88 (0.87–0.89)	0.223			0.266
Endometrioid OC	IVW	280	1.13 (0.97–1.33)	0.121	0.006	0.142	
	MR Egger	280	0.99 (0.78–1.26)	0.942	0.007		
	Weighted median	280	1.00 (0.78–1.27)	0.976			
	MR-PRESSO (raw, 0 outliers)	280	1.13 (1.12–1.14)	0.122			0.005
LMPOC	IVW	280	1.10 (0.95–1.27)	0.193	0.155	0.738	
	MR Egger	280	1.07 (0.86–1.33)	0.541	0.146		
	Weighted median	280	1.05 (0.83–1.33)	0.692			
	MR-PRESSO (raw, 0 outliers)	280	1.10 (1.09–1.11)	0.195			0.159
HGLGSOC	IVW	280	1.04 (0.95–1.13)	0.426	<1 × 10^−3^	0.250	
	MR Egger	280	0.98 (0.85–1.12)	0.738	<1 × 10^−3^		
	Weighted median	280	1.08 (0.95–1.22)	0.248			
	MR-PRESSO (raw, 0 outliers)	280	1.04 (1.03–1.04)	0.427			<1 × 10^−4^
HGSOC	IVW	280	1.02 (0.93–1.12)	0.731	<1 × 10^−3^	0.413	
	MR Egger	280	0.97 (0.84–1.12)	0.700	<1 × 10^−3^		
	Weighted median	280	1.02 (0.89–1.17)	0.795			
	MR-PRESSO (raw, 0 outliers)	280	1.02 (1.01–1.02)	0.731			<1 × 10^−4^
LGSOC	IVW	280	1.43 (1.10–1.86)	0.007	0.015	0.076	
	MR Egger	280	1.10 (0.74–1.62)	0.647	0.020		
	Weighted median	280	1.15 (0.76–1.75)	0.508			
	MR-PRESSO (outlier corrected, 1 outlier)	279	1.45 (1.44–1.47)	0.005			0.017
LGLMSOC	IVW	280	1.28 (1.10–1.48)	0.001	0.185	0.108	
	MR Egger	280	1.11 (0.89–1.39)	0.342	0.205		
	Weighted median	280	1.01 (0.80–1.28)	0.939			
	MR-PRESSO (raw, 0 outliers)	280	1.28 (1.27–1.29)	0.001			0.182
LMSOC	IVW	280	1.22 (1.02–1.44)	0.027	0.470	0.398	
	MR Egger	280	1.12 (0.86–1.45)	0.403	0.466		
	Weighted median	280	1.19 (0.90–1.57)	0.233			
	MR-PRESSO (raw, 0 outliers)	280	1.22 (1.20–1.23)	0.027			0.480
MOC: invasive and low malignant potential	IVW	280	0.99 (0.86–1.15)	0.935	0.478	0.242	
	MR Egger	280	0.90 (0.72–1.12)	0.353	0.484		
	Weighted median	280	0.98 (0.76–1.25)	0.851			
	MR-PRESSO (raw, 0 outliers)	280	0.99 (0.99–1.00)	0.935			0.482
Invasive MOC	IVW	280	1.06 (0.87–1.29)	0.575	0.739	0.032	
	MR Egger	280	0.83 (0.62–1.11)	0.216	0.789		
	Weighted median	280	0.94 (0.69–1.29)	0.707			
	MR-PRESSO (raw, 0 outliers)	280	1.06 (1.05–1.07)	0.564			0.736
LMMOC	IVW	280	0.95 (0.75–1.19)	0.643	0.083	0.569	
	MR Egger	280	1.02 (0.72–1.45)	0.906	0.079		
	Weighted median	280	0.92 (0.64–1.33)	0.671			
	MR-PRESSO (raw, 0 outliers)	280	0.95 (0.93–0.96)	0.644			0.090

Abbreviations: TG, triglycerides; MR, Mendelian randomization; IVW, inverse-variance weighted; OR, odds ratio; CI, confidence interval; SNP, single-nucleotide polymorphism; OC, ovarian cancer; EOC, epithelial ovarian cancer; SOC, serous ovarian cancer; MOC, mucinous ovarian cancer; LMPOC, low malignant potential ovarian cancer; HGLGSOC, high-grade and low-grade serous ovarian cancer; HGSOC, high-grade serous ovarian cancer; LGSOC, low-grade serous ovarian cancer; LGLMSOC, serous ovarian cancer: low-grade and low malignant potential; LMSOC, serous ovarian cancer: low malignant potential; LMMOC, low malignant potential mucinous ovarian cancer.

## Data Availability

All data are available in the submitted manuscript or related sources described in the manuscript.

## Supplementary Materials

The following supporting information can be downloaded at: https://www.mdpi.com/article/10.3390/metabo12121175/s1, Supplementary Table S1: Associations of genetically predicted apolipoprotein A1 (APOA1) with EOC risks in MR analyses; Supplementary Table S2: Associations of genetically predicted apolipoprotein B (APOB) with EOC risks in MR analyses; Supplementary Table S3: Associations of genetically predicted low-density lipoprotein cholesterol (LDL) with EOC risks in MR analyses. Supplementary Dataset S1: Detailed information of the SNPs of the 5 lipids used in the MR analysis on EOC.
