# Supplementary material for: Causal Effects of Circulating Lipid Traits on Epithelial Ovarian Cancer: A Two-Sample Mendelian Randomization Study
Source: Metabolites. 2022 Nov 25;12(12):1175. doi: 10.3390/metabo12121175 (PMC9787029; doi:10.3390/metabo12121175)
Supplement: Supplementary file 1 [file metabolites-12-01175-s001.zip › Causal Role of Lipids in EOC-Supplementary Table 1-Table 3.pdf]

Supplemental materials

Causal Effects of Circulating Lipid Traits on Epithelial Ovarian Cancer: A Two-Sample Mendelian Randomization Study

Contents

Supplemental Table S1. Associations of genetically predicted apolipoprotein A1 (APOA1) with EOC risks in MR analyses……………………………………2

Supplemental Table S2. Associations of genetically predicted apolipoprotein B (APOB) with EOC risks in MR analyses………………………………………6

Supplemental Table S3. Associations of genetically predicted low-density lipoprotein cholesterol (LDL) with EOC risks in MR analyses……………………10

Supplemental Table S1. Associations of genetically predicted apolipoprotein A1 (APOA1) with EOC risks in MR analyses

Main outcome	Method	No. of SNPs	OR (95% CI)	P for association	P for heterogeneity test	P for MR-Egger intercept	P for MR-PRESSO Global test
All EOC	IVW	263	1.02(0.95-1.11)	0.562	<1 x10-3	0.824	
	MR Egger	263	1.01(0.89-1.15)	0.851	<1 x10-3		
	Weighted median	263	0.97(0.87-1.10)	0.666			
	MR-PRESSO (outlier corrected, 1 outlier)	262	1.01(1.01-1.02)	0.722			<1 x10-4
Clear cell OC	IVW	263	1.17(0.93-1.48)	0.176	0.002	0.102	
	MR Egger	263	0.92(0.64-1.33)	0.662	0.002		
	Weighted median	263	1.07(0.76-1.52)	0.692			
	MR-PRESSO (raw, 0 outliers)	263	1.17(1.15-1.18)	0.189			0.001
Endometrioid OC	IVW	263	0.96(0.82-1.13)	0.635	0.061	0.722	
	MR Egger	263	1.00(0.78-1.28)	0.987	0.057		
	Weighted median	263	1.03(0.82-1.30)	0.799			
	MR-PRESSO (raw, 0 outliers)	263	0.95(0.94-0.96)	0.539			0.051
LMPOC	IVW	263	0.87(0.75-1.01)	0.066	0.245	1.000	

	MR Egger	263	0.87(0.69-1.10)	0.251	0.231		
	Weighted median	263	0.81(0.64-1.03)	0.080			
	MR-PRESSO (raw, 0 outliers)	263	0.86(0.86-0.87)	0.053			0.239
HGLGSOC	IVW	263	1.03(0.95-1.13)	0.474	0.001	0.879	
	MR Egger	263	1.02(0.89-1.18)	0.744	0.001		
	Weighted median	263	1.00(0.87-1.15)	0.990			
	MR-PRESSO (outlier corrected, 1 outlier)	262	1.02(1.02-1.02)	0.641			0.002
HGSOC	IVW	263	1.04(0.95-1.15)	0.362	<1 x10-3	0.888	
	MR Egger	263	1.04(0.89-1.20)	0.647	<1 x10-3		
	Weighted median	263	0.99(0.86-1.14)	0.902			
	MR-PRESSO (outlier corrected, 1 outlier)	262	1.03(1.03-1.04)	0.491			<1 x10-3
LGSOC	IVW	263	0.84(0.66-1.08)	0.180	0.384	0.834	
	MR Egger	263	0.82(0.55-1.21)	0.319	0.368		
	Weighted median	263	0.86(0.59-1.26)	0.446			
	MR-PRESSO (raw, 0 outliers)	263	0.84(0.82-0.85)	0.155			0.348

LGLMSOC	IVW	263	0.84(0.73-0.98)	0.022	0.464	0.559	
	MR Egger	263	0.80(0.63-1.01)	0.061	0.453		
	Weighted median	263	0.89(0.70-1.11)	0.296			
	MR-PRESSO (raw, 0 outliers)	263	0.83(0.82-0.84)	0.014			0.361
LMSOC	IVW	263	0.84(0.71-1.01)	0.059	0.648	0.642	
	MR Egger	263	0.80(0.60-1.06)	0.124	0.635		
	Weighted median	263	0.90(0.68-1.20)	0.478			
	MR-PRESSO (raw, 0 outliers)	263	0.83(0.82-0.84)	0.034			0.589
MOC: invasive and low malignant potential	IVW	263	1.04(0.88-1.22)	0.640	0.043	0.117	
	MR Egger	263	1.22(0.94-1.59)	0.130	0.050		
	Weighted median	263	1.15(0.88-1.51)	0.296			
	MR-PRESSO (raw, 0 outliers)	263	1.06(1.05-1.07)	0.480			0.042
Invasive MOC	IVW	263	1.15(0.93-1.42)	0.185	0.193	0.078	
	MR Egger	263	1.45(1.04-2.03)	0.028	0.221		
	Weighted median	263	1.23(0.86-1.74)	0.258			

	MR-PRESSO (raw, 0 outliers)	263	1.18(1.17-1.20)	0.113			0.196
LMMOC	IVW	263	0.91(0.72-1.14)	0.393	0.337	0.538	
	MR Egger	263	0.99(0.69-1.42)	0.957	0.328		
	Weighted median	263	1.03(0.70-1.52)	0.887			
	MR-PRESSO (raw, 0 outliers)	263	0.92(0.90-0.93)	0.445			0.354

Abbreviations: APOA1, apolipoprotein A1; MR, mendelian randomization; IVW, inverse-variance weighted; OR, odds ratio; CI, confidence interval; SNP, single-nucleotide polymorphism; OC, ovarian cancer; EOC, epithelial ovarian cancer; SOC, serous ovarian cancer; MOC, mucinous ovarian cancer; LMPOC, low malignant potential ovarian cancer; HGLGSOC, high-grade and low-grade serous ovarian cancer; HGSOC, high-grade serous ovarian cancer; LGSOC, low-grade serous ovarian cancer; LGLMSOC, serous ovarian cancer: low-grade and low malignant potential; LMSOC, serous ovarian cancer: low malignant potential; LMMOC, low malignant potential mucinous ovarian cancer.

Supplemental Table S2. Associations of genetically predicted apolipoprotein B (APOB) with EOC risks in MR analyses.

Main outcome	Method	No. of SNPs	OR (95% CI)	P for association	P for heterogeneity test	P for MR-Egger intercept	P for MR-PRESSO Global test
All EOC	IVW	176	1.03(0.95-1.11)	0.461	0.001	0.001	
	MR Egger	176	1.00(0.90-1.11)	0.959	0.001		
	Weighted median	176	1.04(0.93-1.15)	0.512			
	MR-PRESSO (raw, 0 outliers)	176	1.03(1.02-1.04)	0.436			0.001
Clear cell OC	IVW	176	1.01(0.82-1.25)	0.895	0.173	0.836	
	MR Egger	176	0.99(0.75-1.32)	0.965	0.161		
	Weighted median	176	1.15(0.84-1.57)	0.395			
	MR-PRESSO (raw, 0 outliers)	176	1.02(1.00-1.04)	0.856			0.170
Endometrioid OC	IVW	176	1.06(0.90-1.24)	0.516	0.002	0.734	
	MR Egger	176	1.03(0.82-1.28)	0.805	0.001		
	Weighted median	176	0.95(0.74-1.21)	0.660			
	MR-PRESSO (raw, 0 outliers)	176	1.05(1.04-1.07)	0.528			0.002
LMPOC	IVW	176	0.92(0.80-1.07)	0.271	0.262	0.502	

	MR Egger	176	0.88(0.72-1.07)	0.208	0.252		
	Weighted median	176	0.94(0.76-1.16)	0.549			
	MR-PRESSO (raw, 0 outliers)	176	0.92(0.91-0.94)	0.286			0.277
HGLGSOC	IVW	176	1.01(0.93-1.10)	0.773	0.006	0.279	
	MR Egger	176	0.97(0.86-1.09)	0.601	0.006		
	Weighted median	176	1.00(0.89-1.13)	0.980			
	MR-PRESSO (raw, 0 outliers)	176	1.01(1.01-1.02)	0.757			0.007
HGSOC	IVW	176	1.01(0.93-1.11)	0.785	0.004	0.330	
	MR Egger	176	0.97(0.86-1.10)	0.644	0.004		
	Weighted median	176	1.04(0.92-1.17)	0.558			
	MR-PRESSO (raw, 0 outliers)	176	1.01(1.01-1.02)	0.771			0.006
LGSOC	IVW	176	1.08(0.84-1.38)	0.558	0.096	0.738	
	MR Egger	176	1.04(0.74-1.45)	0.836	0.088		
	Weighted median	176	0.98(0.67-1.43)	0.929			
	MR-PRESSO (raw, 0 outliers)	176	1.08(1.06-1.10)	0.545			0.109

LGLMSOC	IVW	176	1.03(0.89-1.19)	0.683	0.286	0.199	
	MR Egger	176	0.94(0.78-1.15)	0.570	0.299		
	Weighted median	176	0.95(0.75-1.19)	0.640			
	MR-PRESSO (raw, 0 outliers)	176	1.03(1.02-1.04)	0.687			0.319
LMSOC	IVW	176	1.01(0.85-1.20)	0.881	0.838	0.284	
	MR Egger	176	0.93(0.73-1.17)	0.537	0.841		
	Weighted median	176	0.98(0.75-1.28)	0.888			
	MR-PRESSO (raw, 0 outliers)	176	1.01(1.00-1.02)	0.892			0.857
MOC: invasive and low malignant potential	IVW	176	0.95(0.80-1.12)	0.530	0.021	0.307	
	MR Egger	176	1.03(0.82-1.29)	0.814	0.021		
	Weighted median	176	1.08(0.85-1.36)	0.545			
	MR-PRESSO (raw, 0 outliers)	176	0.95(0.94-0.97)	0.587			0.015
Invasive MOC	IVW	176	1.09(0.87-1.36)	0.447	0.008	0.230	
	MR Egger	176	1.24(0.91-1.68)	0.170	0.009		
	Weighted median	176	1.25(0.89-1.75)	0.191			

	MR-PRESSO (raw, 0 outliers)	176	1.09(1.07-1.11)	0.431			0.009
LMMOC	IVW	176	0.78(0.61-0.99)	0.038	0.041	0.823	
	MR Egger	176	0.80(0.58-1.10)	0.172	0.037		
	Weighted median	176	0.90(0.63-1.29)	0.570			
	MR-PRESSO (outlier corrected, 1 outlier)	175	0.77(0.76-0.79)	0.030			0.026

Abbreviations: APOB, apolipoprotein B; MR, mendelian randomization; IVW, inverse-variance weighted; OR, odds ratio; CI, confidence interval; SNP, single-nucleotide polymorphism; OC, ovarian cancer; EOC, epithelial ovarian cancer; SOC, serous ovarian cancer; MOC, mucinous ovarian cancer; LMPOC, low malignant potential ovarian cancer; HGLGSOC, high-grade and low-grade serous ovarian cancer; HGSOC, high-grade serous ovarian cancer; LGSOC, low-grade serous ovarian cancer; LGLMSOC, serous ovarian cancer: low-grade and low malignant potential; LMSOC, serous ovarian cancer: low malignant potential; LMMOC, low malignant potential mucinous ovarian cancer.

Supplemental Table S3. Associations of genetically predicted low-density lipoprotein cholesterol (LDL) with EOC risks in MR analyses.

Main outcome	Method	No. of SNPs	OR (95% CI)	P for association	P for heterogeneity test	P for MR-Egger intercept	P for MR-PRESSO Global test
All EOC	IVW	158	1.04(0.95-1.14)	0.433	<1 x10-3	0.553	
	MR Egger	158	1.01(0.88-1.15)	0.927	<1 x10-3		
	Weighted median	158	1.03(0.91-1.16)	0.617			
	MR-PRESSO (raw, 0 outliers)	158	1.04(1.03-1.05)	0.434			<1 x10-3
Clear cell OC	IVW	158	0.99(0.79-1.26)	0.963	0.760	0.673	
	MR Egger	158	0.94(0.67-1.33)	0.732	0.746		
	Weighted median	158	1.07(0.74-1.57)	0.707			
	MR-PRESSO (raw, 0 outliers)	158	0.99(0.98-1.01)	0.961			0.772
Endometrioid OC	IVW	158	1.12(0.92-1.36)	0.247	0.001	0.323	
	MR Egger	158	1.01(0.76-1.35)	0.956	0.001		
	Weighted median	158	1.03(0.79-1.35)	0.803			
	MR-PRESSO (raw, 0 outliers)	158	1.12(1.10-1.14)	0.249			0.001

LMPOC	IVW	158	0.90(0.77-1.07)	0.230	0.599	0.918	
	MR Egger	158	0.90(0.71-1.14)	0.375	0.577		
	Weighted median	158	0.91(0.71-1.17)	0.471			
	MR-PRESSO (raw, 0 outliers)	158	0.90(0.89-0.92)	0.224			0.607
HGLGSOC	IVW	158	1.03(0.93-1.13)	0.632	0.006	0.357	
	MR Egger	158	0.97(0.84-1.13)	0.723	0.006		
	Weighted median	158	1.00(0.87-1.14)	0.945			
	MR-PRESSO (raw, 0 outliers)	158	1.03(1.02-1.03)	0.633			0.009
HGSOC	IVW	158	1.03(0.93-1.15)	0.539	0.002	0.336	
	MR Egger	158	0.98(0.83-1.14)	0.769	0.002		
	Weighted median	158	1.03(0.90-1.19)	0.637			
	MR-PRESSO (raw, 0 outliers)	158	1.03(1.03-1.04)	0.540			0.002
LGSOC	IVW	158	0.96(0.72-1.28)	0.778	0.261	0.864	
	MR Egger	158	0.99(0.65-1.50)	0.948	0.243		
	Weighted median	158	0.85(0.55-1.31)	0.453			

	MR-PRESSO (raw, 0 outliers)	158	0.96(0.94-0.98)	0.778			0.273
LGLMSOC	IVW	158	1.00(0.84-1.18)	0.961	0.216	0.714	
	MR Egger	158	0.96(0.74-1.24)	0.762	0.202		
	Weighted median	158	0.93(0.72-1.21)	0.604			
	MR-PRESSO (raw, 0 outliers)	158	1.00(0.98-1.01)	0.961			0.232
LMSOC	IVW	158	1.02(0.83-1.24)	0.875	0.810	0.531	
	MR Egger	158	0.95(0.70-1.27)	0.723	0.800		
	Weighted median	158	0.90(0.65-1.24)	0.512			
	MR-PRESSO (raw, 0 outliers)	158	1.02(1.00-1.03)	0.869			0.815
MOC: invasive and low malignant potential	IVW	158	0.91(0.75-1.11)	0.341	0.015	0.192	
	MR Egger	158	1.05(0.79-1.40)	0.751	0.017		
	Weighted median	158	1.09(0.82-1.44)	0.565			
	MR-PRESSO (raw, 0 outliers)	158	0.91(0.90-0.92)	0.342			0.016
Invasive MOC	IVW	158	1.05(0.81-1.36)	0.696	0.028	0.174	
	MR Egger	158	1.28(0.88-1.87)	0.205	0.032		

	Weighted median	158	1.20(0.81-1.77)	0.368			
	MR-PRESSO (raw, 0 outliers)	158	1.05(1.03-1.07)	0.697			0.028
LMMOC	IVW	158	0.74(0.56-0.97)	0.030	0.054	0.590	
	MR Egger	158	0.80(0.53-1.20)	0.282	0.050		
	Weighted median	158	0.66(0.43-1.01)	0.053			
	MR-PRESSO (raw, 0 outliers)	158	0.74(0.72-0.75)	0.031			0.050

Abbreviations: LDL, low-density lipoprotein cholesterol; MR, mendelian randomization; IVW, inverse-variance weighted; OR, odds ratio; CI, confidence interval; SNP, single-nucleotide polymorphism; OC, ovarian cancer; EOC, epithelial ovarian cancer; SOC, serous ovarian cancer; MOC, mucinous ovarian cancer; LMPOC, low malignant potential ovarian cancer; HGLGSOC, high-grade and low-grade serous ovarian cancer; HGSOC, high-grade serous ovarian cancer; LGSOC, low-grade serous ovarian cancer; LGLMSOC, serous ovarian cancer: low-grade and low malignant potential; LMSOC, serous ovarian cancer: low malignant potential; LMMOC, low malignant potential mucinous ovarian cancer.
